# Prevalence of Pulmonary Hypertension among Sudanese Patients with Sickle Cell Disease

**DOI:** 10.2174/0118743064292252240422100911

**Published:** 2024-05-21

**Authors:** Yousif Ahmed Elfaki, Ahmed Saadeldin Ibrahim, Tarig Hakim Merghani

**Affiliations:** 1 Department of Internal Medicine, Omdurman Teaching Hospital, Omdurman, Sudan; 2 Department of Medicine, Prince Sultan Military Medical City, Riyadh, KSA; 3 Department of Physiology, RAK College of Medical Sciences, RAK Medical and Health Sciences University, RAK, UAE

**Keywords:** Pulmonary hypertension, Sickle cell disease, Electrophoresis, Cigarette smoking, Hydroxyurea, Sudanese

## Abstract

**Background:**

Sickle Cell Disease (SCD) is a hereditary condition characterized by aberrant red blood cell morphology, leading to persistent hemolytic anemia. The consequential impact of SCD on the pulmonary vasculature can result in pulmonary hypertension (PHT), a severe complication that detrimentally affects the well-being and survival of individuals with SCD. The prevalence and risk determinants of PHT in SCD patients exhibit variations across diverse geographical regions and populations. This study aims to ascertain the prevalence of PHT among Sudanese SCD patients and identify associated factors.

**Methods:**

A cohort of thirty-one adult sickle cell disease (SCD) patients, as confirmed by hemoglobin electrophoresis, were recruited for participation in this cross-sectional study. Comprehensive data encompassing demographic, clinical, and laboratory parameters were collected. Doppler echocardiography was employed to quantify pulmonary arterial systolic pressure (PASP) and evaluate right ventricular size and function.

**Results:**

Within our cohort, the prevalence of PHT was 29%. Active cigarette smoking demonstrated a significant association with PHT (P=0.042), while hydroxyurea therapy exhibited no noticeable impact on PHT (P=0.612).

**Conclusion:**

Our investigation revealed a PHT prevalence of less than one-third in our SCD patient population, aligning with prior studies. Notably, independent of other factors, cigarette smoking emerged as a distinct risk factor for PHT in SCD patients. This highlights the potential utility of smoking cessation as an intervention to delay the onset of this condition. However, further research is imperative to elucidate the mechanisms through which smoking contributes to PHT development in individuals with SCD.

## 1 INTRODUCTION

Sickle Cell Disease (SCD) is a hereditary condition resulting from a single-point mutation in the β-globin gene, leading to the production of abnormal hemoglobin S (HbS) [1]. Its prevalence is significant, particularly in regions, such as sub-Saharan Africa, India, Saudi Arabia, and South America. Homozygosity for the sickle hemoglobin (HbS) gene mutation characterizes individuals with SCD, inducing hemolytic anemia and complications, including vaso-occlusive pain crises (VOC) and acute chest syndrome [1, 2]. Pathological manifestations typically emerge within the first year of life due to hemoglobin S precipitation during deoxygenation, resulting in sickle-shaped erythrocytes, impeding blood flow, and causing hemolysis and vaso-occlusive episodes.

SCD patients face the risk of progressive vasculopathy marked by systemic and pulmonary hypertension (PHT), endothelial dysfunction, and proliferative changes in blood vessel intima and smooth muscle [1]. Chronic hemolysis of sickle cells is associated with mild to moderate anemia, reticulocytosis, unconjugated hyper- bilirubinemia, elevated serum lactate dehydrogenase, and diminished serum haptoglobin. Heterozygous individuals (sickle cell trait) exhibit less severe anemia; however, they often demonstrate subtle changes in laboratory parameters reflective of chronic hemolysis. These may include mild elevation in reticulocyte count, unconjugated hyper- bilirubinemia, elevated serum lactate dehydrogenase, and a reduction in serum haptoglobin.

Vaso-occlusive crises represent the primary cause of acute and chronic multisystem failure associated with SCD. While acute complications may lead to premature mortality, advancements in supportive care have markedly contributed to an enhanced life expectancy for individuals with sickle cell disease (SCD), extending survival well beyond the fifth decade [2]. The implementation of comprehensive healthcare strategies, including disease-modifying therapies, blood transfusions, pain management, and preventive measures, has played a pivotal role in mitigating complications and improving the overall quality of life for individuals with SCD.

Pulmonary hypertension (PHT) emerges as a severe and potentially fatal complication of SCD, adversely affecting pulmonary vessels and the structure and function of the right ventricle. Pulmonary hypertension (PHT) is characterized by a mean pulmonary artery pressure exceeding 25 mmHg at rest. Despite its clinical significance, the prevalence and risk factors of PHT in sickle cell disease (SCD) patients remain inadequately defined, exhibiting variations across studies and populations. Previous investigations have highlighted a notably high prevalence of moderate to severe PHT in African patients [3, 4]. The presence of PHT in individuals with SCD is closely linked to a cascade of debilitating consequences, encompassing not only diminished exercise capacity, compromised quality of life, and a pronounced impact on daily functioning but also a substantial reduction in survival rates [5]. Clinical manifestations encompass dyspnea, fatigue, chest pain, and peripheral edema, with physical examination revealing signs indicative of right ventricular abnormalities that include a loud second heart sound, right-sided fourth heart sound, right ventricular hypertrophy, and right parasternal heave. These signs, by offering valuable insights into the structural and functional alterations occurring within the cardiovascular system, not only aid in the diagnosis of PHT but also guide the formulation of targeted management plans.

Diagnosing PHT in SCD patients traditionally involves invasive measurement of pulmonary artery pressure through right heart catheterization, which is recognized as the gold standard method. However, this procedure faces limitations in resource-limited settings, such as sub-Saharan Africa and Sudan, where SCD is highly prevalent. Consequently, non-invasive modalities, particularly Doppler echocardiography, are frequently employed for estimating Pulmonary Artery Systolic Pressure (PASP) and evaluating right ventricular size and function [6]. Doppler echocardiography serves as a valuable screening tool for identifying individuals at risk of developing PHT, guiding further assessment and intervention. Acute sickle cell events, such as vaso-occlusive crisis and acute chest syndrome, can exacerbate PHT in SCD patients, leading to sudden deterioration and potentially fatal outcomes [7]. Consequently, the timely detection and management of PHT in SCD patients are imperative for improving their overall outcomes and prognosis.

This study employs a comprehensive approach to investigate the prevalence and characteristics of PHT in adult Sudanese SCD patients through the utilization of Doppler echocardiography. Beyond establishing the prevalence of PHT, our investigation seeks to identify associated factors contributing to the manifestation of this complication in the studied population.

## 2 METHODS

### 2.1 Study Design and Setting

This is a cross-sectional hospital-based study. It was carried out in the Hematology Department of Omdurman Military Hospital in Khartoum, Sudan. Ethical approval and permission to conduct the study were obtained from the Ethical Committee of the Hospital (REC-OMH, 018). The participation of all patients in the study was dependent upon their informed written consent, which was obtained before the initiation of data collection. The study adheres to the ethical principles governing medical research involving human subjects, as delineated in the Helsinki Declaration.

### 2.2 Study Population and Sampling

During the study period, we approached all identifiable adult patients diagnosed with sickle cell anemia who sought medical attention at the hospital. The confirmation of SCD for each participant was established through hemoglobin electrophoresis. Exclusions comprised individuals aged 17 years or younger, as well as those presenting with potential alternative causes of pulmonary hypertension (PHT), including valvular lesions, chronic lung diseases, or human immunodeficiency virus infection.

### 2.3 Methods of Data Collection

Each participant responded to an interviewer-based questionnaire that collects information on the patient’s age, gender, smoking, current symptoms, history of acute and chronic complications of SCD, and the utilization of hydroxyurea as a therapeutic intervention. Echocardio- graphy was used to estimate the pulmonary arterial systolic pressure (PASP) and assess the right ventricular size and function. Tricuspid regurgitation was identified in the best possible way for proper continuous wave Doppler alignment. The right atrium/right ventricle pressure gradient (RA/RVPG) was calculated using a simplified Bernoulli’s equation (4V^2^) [8].

### 2.4 Statistical Analyses

Descriptive data analysis was performed using the Statistical Package for the Social Sciences (SPSS) software version 20.0 (IBM Corp., Armonk, NY, USA). Categorical variables were expressed as frequencies and percentages and subjected to comparison through the chi-square test. A level of statistical significance was acknowledged for p-values less than 0.05.

## 3 RESULTS

Table **1** presents the demographic characteristics of the participants. The study included 31 SCD patients, of whom 16 (51.6%) were females. The mean age of the participants was 24.4 ± 5.2 years, ranging from 18 to 38 years. Most participants (26, 83.9%) were 30 years old or younger. The majority of the participants (90.3%) had the HbSS genotype, followed by two (6.5%) with the HbSA genotype and one (3.2%) with the HbS/Thal genotype. All participants belonged to either poor (14, 45.2%) or moderate (17, 54.8%) socioeconomic status.

Table **2** delineates the presenting symptoms and complications observed in individuals with sickle cell disease (SCD) during the study period. Notably, nearly half of the participants (48.4%) presented as asymptomatic. Among those experiencing symptoms, exertional dyspnea was reported by 29%, while chest pain and palpitation were noted in 9.7% and 3.2% of the cases, respectively.

A detailed exploration of the participants' medical history revealed that aplastic crisis was reported in 51.6% of individuals, painful crisis in 83.9%, sequestration crisis in 19.4%, stroke in 9.7%, and acute chest syndrome in 54.8%.

Moreover, chronic conditions associated with SCD were prevalent among the participants, with gallstones affecting 38.7%, chronic kidney disease present in 6.5%, chronic leg ulcers observed in 32.3%, retinal disease in 9.7%, and avascular necrosis in 35.5% of cases.

The echocardiographic assessment of pulmonary arterial systolic pressure (PASP) revealed elevated values in 29% of the participants, ranging from 41 to 85 mmHg (Table **3**). Notably, high PASP showed no significant association with gender or the administration of hydroxyurea as part of the treatment protocol.

Among the study participants, approximately two-thirds of active smokers and fewer than half of passive smokers exhibited pulmonary hypertension (PHT), in contrast to only 11% of non-smokers. This observation underscored a statistically significant relationship between cigarette smoking and the occurrence of PHT (p= 0.042). These findings highlight the potential role of cigarette smoking as a significant factor in the development of pulmonary hypertension in individuals with sickle cell disease.

## 4 DISCUSSION

Given that sickle cell disease (SCD) is an autosomal recessive disorder, there exists no obvious gender disparity in its prevalence. In our study, the male-to-female ratio closely approximates 1:1, underscoring the neutral distribution of the disease across genders. The age range of participants spanned from 18 to 38 years, with a mean age of 24.4 ± 5.2 years. A noteworthy majority of participants (83.9%) were aged 30 years or younger, and none reached the age of 40. This demographic distribution reflects the relatively truncated life expectancy of individuals with SCD in developing countries, where access to supportive care for severe complications remains limited. It is essential to highlight that SCD patients of African American descent in the United States may exhibit prolonged survival; however, their adherence to treatment regimens is often compromised, likely due to economic burdens [9]. A recent investigation into SCD-related mortality trends in the United States revealed a median age at death of 43 years, with most fatalities attributed to the long-term complications of the disease [2]. This disparity highlights the impact of socioeconomic factors on the management and outcomes of SCD, emphasizing the need for targeted interventions to improve access to comprehensive care and enhance the life expectancy of affected individuals.

The prevailing genotype among the majority of our participants was HbSS (sickle cell disease), encompassing 90.3% of the cohort. Only two participants (6.5%) exhibited the HbSA genotype, commonly recognized as sickle cell trait, and one participant (3.2%) presented with the HbS/Thal genotype, representing a combination of sickle cell and beta-thalassemia genes. These genetic distributions align with the established global prevalence patterns of sickle cell disease genotypes. Specifically, the predominance of HbSS in our study cohort mirrors its prevalence in the Americas, the United Kingdom, and Africa. Conversely, the presence of the HbS/Thal genotype, although less frequent, is consistent with higher occurrences reported in populations, such as Greece and India [10]. This genetic heterogeneity emphasizes the importance of understanding regional variations in the distribution of sickle cell disease genotypes for effective disease management and genetic counseling.

The clinical presentation and complications observed in our study yielded an unexpected finding, with half of the participants (50%) being asymptomatic, a discrepancy that appears inconsistent with the known severity of sickle cell disease (SCD). It is noteworthy that symptoms may not always align with the overall disease severity, as patients might adapt to their condition or underreport their complaints. Conversely, in a large multicenter study encompassing SCD patients globally, only 2% of participants were asymptomatic for one month before the study. In that study, most patients reported symptoms, such as fatigue (65%), bone aches (51%), and headache (47%) [11]. In our study, the reported symptoms included exertional dyspnea (29%), chest pain (9.7%), and palpitation (3.2%). In a recent study conducted among children suffering from SCD in Sudan, the major presenting symptoms included fever (48%), cough (20%), fatigability (14%), and shortness of breath (14%) [12]. Manifestations of anemia-like pallor appeared in 62% of the children [12].

A recent investigation into primary and associated complications in individuals with SCD revealed a high prevalence of vaso-occlusive crisis history (85%), pulmonary disorders (76.1%), infectious diseases (55.1%), avascular bone necrosis (29.7%), and cerebrovascular conditions (20.5%) [13]. In our cohort, a history of painful crisis was reported in 83.9%, while stroke affected 9.7%, acute chest syndrome 54.8%, chronic kidney disease 6.5%, chronic leg ulcers 32.3%, retinal disease 9.7%, and avascular necrosis 35.5%. These complications are likely attributed to vasculopathy and acute infarctions, contributing to the complex clinical landscape of SCD.

Our echocardiographic assessment revealed features indicative of pulmonary hypertension (PHT) in 29% of the participants, demonstrating a pulmonary arterial systolic pressure (PASP) range of 41 to 85 mmHg. These findings align with the prevalence reported in prior studies that employed echocardiography to evaluate PHT among individuals with sickle cell disease (SCD) [14, 15]. A regional investigation conducted in Nigeria reported a comparable prevalence of 25% of PHT in SCD patients [3]. Additionally, a substantial screening study utilizing Doppler echocardiography in patients with homozygous SS disease in the United States and England found that elevated PASP was associated with more severe hemolytic anemia and renal insufficiency [16].

The precise mechanism underlying the development of PHT in SCD remains incompletely understood, although chronic hemolysis, endothelial dysfunction, inflammation, and thrombosis are implicated. While the current classification categorizes PHT in SCD as group 1 (pulmonary arterial hypertension), some researchers have suggested its consideration within group 5 (unclear or multifactorial mechanisms) [17]. Notably, PHT in SCD is linked to diminished exercise capacity, compromised quality of life, and reduced survival, indicating the imperative for early detection and intervention in its management.

Hydroxyurea stands as a widely successful treatment for sickle cell disease (SCD) patients globally, operating by augmenting the production of fetal hemoglobin (HbF). This increase in HbF levels mitigates the sickling of red blood cells and reduces the frequency of vaso-occlusive crises [18]. However, in our cohort, where a substantial proportion of patients presented as asymptomatic, the initiation of specific treatment with hydroxyurea was not deemed necessary.

Upon subjecting those who received hydroxyurea treatment to statistical analysis, the results indicated an insignificant effect on pulmonary hypertension (PHT). Potential explanations for this outcome may include the relatively small sample size, the administration of a low hydroxyurea dosage, or potential challenges with treatment adherence. To comprehensively evaluate the efficacy and safety of hydroxyurea therapy for PHT in individuals with SCD, further studies with larger sample sizes and meticulous monitoring of treatment adherence are imperative. These endeavors will contribute invaluable insights into optimizing the management of PHT in SCD patients, advancing our understanding of the therapeutic potential of hydroxyurea in this context.

Our study uncovered a notable prevalence of pulmonary hypertension (PHT) among sickle cell disease (SCD) patients who actively smoked cigarettes, showing a substantial contrast to those who did not smoke or were exposed to passive smoking. This observation reflects a potential dose-dependent effect of tobacco smoke in elevating pulmonary artery pressure. The consistency of our findings with prior studies reinforces the well-established association between cigarette smoking and the development of PHT [19, 20]. It is conceivable that the inhalation of toxins and subsequent hypoxia induced by chronic cigarette smoking may exacerbate the severity of pulmonary hypertension (PHT); nevertheless, additional research is warranted to validate this hypothesis.

These results highlight the imperative for preventive measures targeting PHT in individuals with SCD, particularly involving educational sessions emphasizing the detrimental impact of cigarette smoking on their health. Incorporating comprehensive smoking cessation programs within the care framework for SCD patients may be useful in modifying the risk of PHT and improving overall health outcomes in this vulnerable population.

## CONCLUSION

In conclusion, our study revealed a prevalence of pulmonary hypertension (PHT) in less than one-third of our sickle cell disease (SCD) participants. Notably, we identified a significant association between cigarette smoking and the development of pulmonary hypertension in individuals with SCD. The primary limitation of our study lies in its modest sample size, a constraint that could be addressed through the extension of the study duration. Furthermore, the exclusion of chronic lung disease was not solely determined by spirometry results and oxygen saturation measurements, potentially leaving room for other causes of pulmonary hypertension (PHT). Additionally, the lack of follow-up data restricts our ability to attain a thorough understanding of the morbidity and mortality outcomes associated with PHT in patients with sickle cell disease (SCD), while potential recall bias remains a significant concern.

Despite these limitations, our findings advocate for the utility of Doppler echocardiography in screening adult SCD patients for PHT. By employing this non-invasive modality, we aim to overcome the challenges associated with the limited availability and feasibility of right heart catheterization in resource-constrained settings prevalent in Sudan. Moreover, it is suggested that smoking cessation holds promise as an intervention to potentially prevent or delay the onset of this condition. To further advance our understanding of PHT-related morbidity and mortality in African countries, we strongly recommend additional comprehensive studies with larger sample sizes and extended follow-up periods. Such investigations will contribute crucial information for refining clinical practices and tailoring interventions to optimize the management of PHT in individuals with SCD in this population.

## Figures and Tables

**Table 1:** Demographic characteristics of the participants.

Parameter		Frequency n (%)
**Gender**	Male	15 (48.4%)
	Female	16 (51.6%)
**Age groups (y)**	18-23	13 (41.9%)
	24-30	13 (41.9%)
	> 31	5 (16.2%)
**Hemoglobin genotype**	HbSS	28 (90.3%)
	HbAS	2 (6.5%)
	HbS/Thal	1 (3.2%)
**Economic status**	Poor	14 (45.2%)
	Moderate	17 (54.8%)

**Table 2:** History of acute and chronic symptoms and complications among patients with sickle cell disease.

Parameter		Frequency n (%)
**Symptoms**	Asymptomatic	15 (48.4%)
	Exertional dyspnea	9 (29%)
	Recurrent chest pain	3 (9.7%)
	Exertional dyspnea with palpitation	1 (3.2%)
**SCD History of acute complications**	Aplastic crisis	16 (51.6%)
	Painful crisis	26 (83.9%)
	Stroke	3 (9.7%)
	Sequestration crisis	6 (19.4%)
	Acute chest syndrome	17 (54.8%)
**SCD Chronic complications**	Gallstones	12 (38.7%)
	Chronic renal disease	2 (6.5%)
	Chronic leg ulcer	10 (32.3%)
	Retinal disease	3 (9.7%)
	Avascular necrosis	11 (35.5%)

**Table 3:** Pulmonary arterial systolic pressure (PASP) in relation to gender, treatment with hydroxyurea, and smoking among the participants.

-		Pulmonary Arterial Systolic Pressure (PASP)		-	-
Parameter		Normal	Pulmonary Hypertension	Total	P-value
**Gender**	Female	11 (73.3%)	4 (26.7%)	15 (100%)	0.779
	Male	11 (68.8%)	5 (31.2%)	16 (100%)	
	Total	22 (71.0%)	9 (29.0%)	31 (100%)	
**Use of hydroxyurea**	Not used	12 (63.2%)	7 (36.8%)	19 (100%)	0.628
	≤ 6 months	3 (75.0%)	1 (25.0%)	4 (100%)	
	≥ 6 months	7 (87.5%)	1 (12.5%)	8 (100%)	
	Total	22 (71.0%)	9 (29.0%)	31 (100%)	
**Smoking**	None	16 (88.9%)	2 (11.1%)	18 (100%)	0.042
	Passive	4 (57.1%)	3 (42.9%)	7 (100%)	
	Active	2 (33.3%)	4 (66.7%)	6 (100%)	
	Total	22 (71.0%)	9 (29.0%)	31 (100%)	

## Data Availability

The data sets used and/or analysed during this study are available from the corresponding author upon request.

## LIST OF ABBREVIATIONS

SCD: Sickle Cell Disease
PHT: Pulmonary Hypertension
PASP: Pulmonary Arterial Systolic Pressure

## STANDARDS OF REPORTING

STROBE guidelines were followed.
